# Anti-vascular endothelial growth factor agent reduces inflammation in macular edema with central retinal vein occlusion

**DOI:** 10.1186/s12950-019-0214-2

**Published:** 2019-05-22

**Authors:** Asako Mashima, Hidetaka Noma, Kanako Yasuda, Hiroshi Goto, Masahiko Shimura

**Affiliations:** 1 Department of Ophthalmology, Hachioji Medical Center, Tokyo Medical University, 1163, Tatemachi, Hachioji, Tokyo 193-0998 Japan; 2 Department of Ophthalmology, Tokyo Medical University, Tokyo, Japan

## Abstract

**Background:**

Correlations among the aqueous flare value (an indicator of inflammation), functional-morphologic parameters, and aqueous humor levels of growth factors/receptors and inflammatory factors/cytokines were investigated in patients with central retinal vein occlusion (CRVO) and macular edema who received intravitreal ranibizumab injection (IRI) and were followed for 6 months.

**Methods:**

Aqueous humor levels of 11 cytokines or growth inflammatory/factors were measured in 20 CRVO patients with macular edema receiving IRI. Patients with recurrent macular edema were administered further IRI as needed. Aqueous humor levels of vascular endothelial growth factor (VEGF), soluble VEGF receptor (sVEGFR), and other cytokines/inflammatory factors were measured by the suspension array method. Aqueous flare values were measured with a laser flare meter and macular edema was examined by optical coherence tomography.

**Results:**

Compared with before treatment (baseline), the aqueous flare value showed a significant decrease at both 1 month and 6 months after IRI therapy. There were significant correlations between the aqueous flare value and the aqueous levels of sVEGFR-1, placental growth factor, monocyte chemoattractant protein 1, soluble intercellular adhesion molecule-1, interleukin (IL)-6, and IL-8. In addition, a significant correlation was noted between the change of the aqueous flare value and improvement of central macular thickness at 6 months after IRI, as well as a significant correlation between the change of the aqueous flare value and improvement of best-corrected visual acuity at 6 months.

**Conclusions:**

These findings suggest that IRI reduces inflammation and that the aqueous flare value is influenced by inflammatory factors/cytokines. In addition, the change of the aqueous flare value may be an indicator of the long-term prognosis in CRVO patients receiving IRI therapy for macular edema.

## Background

Central retinal vein occlusion (CRVO) is a common retinal vascular disease. Macular edema is frequent in CRVO patients and is the main reason they develop visual impairment. Occlusion of retinal vessels leads to bleeding and accumulation of serous fluid, followed by edema and inflammation. In patients with CRVO, macular edema is often treated by intravitreal injection of ranibizumab, which is an Fab antibody fragment that binds and neutralizes all isoforms of vascular endothelial growth factor (VEGF)-A, or by aflibercept, which is a recombinant fusion protein containing parts of the human VEGF receptor (VEGFR)-1 and VEGFR-2 extracellular domains fused to the Fc portion of human immunoglobulin G1 [1, 2]. However, macular edema persists in some patients despite administration of ranibizumab or aflibercept [1, 2], suggesting the involvement of other vasoactive factors along with VEGF.

We recently reported that the intraocular levels of several inflammatory factors were significantly correlated with the severity of macular edema in CRVO [3, 4], suggesting an important role of inflammation in the mechanism of macular edema. This hypothesis is supported by the results of the Standard Care vs Corticosteroid for Retinal Vein Occlusion (SCORE) study, which demonstrated improvement of both visual acuity and macular edema in CRVO patients at 12 months after intravitreal injection of triamcinolone acetonide [5]. It was also reported that the vitreous fluid levels of VEGF, soluble intercellular adhesion molecule-1 (sICAM-1), and interleukin (IL)-6 are significantly correlated with the aqueous flare value (an indicator of inflammation) in patients with CRVO [6]. However, the relation between changes of the aqueous flare value and the prognosis of CRVO after anti-VEGF therapy remains unclear. Accordingly, we evaluated the relations among the aqueous flare value, functional-morphologic parameters, and aqueous humor levels of cytokines/growth factors in CRVO patients receiving anti-VEGF agents for macular edema.

## Methods

### Subjects

Between August 2013 and October 2015, 20 CRVO patients received intravitreal ranibizumab injection (IRI) at a dose of 0.5 mg in 0.05 ml (Lucentis; Novartis, Buläch, Switzerland). Criteria for IRI therapy were a central macular thickness (CMT) > 300 μm and best-corrected visual acuity (BCVA) < 25/30. None of the patients had received treatment for macular edema before this study. After each patient gave informed consent, the aqueous flare was measured with a laser flare meter (FC-600, Kowa Co. Ltd., Tokyo, Japan), as described previously [6]. At the time of IRI, a mean volume of 0.1 mL of aqueous humor was collected by anterior chamber limbal paracentesis with a 30-gauge needle attached to an insulin syringe. The aqueous humor levels of cytokines (sVEGFR-1, sVEGFR-2, VEGF, placental growth factor (PlGF), platelet-derived growth factor (PDGF)-AA, sICAM-1, monocyte chemoattractant protein 1 (MCP-1), interleukin (IL)-6, IL-8, IL-12(p70), and IL-13) were measured with enzyme-linked immunosorbent assays (xMAP; Luminex Corp. Austin, TX), as reported previously [4]. The patients were evaluated at 1-month intervals after IRI by complete eye examination, spectral-domain optical coherence tomography (OCT; Heidelberg Engineering, Heidelberg, Germany), measurement of vital signs, review of the medical history (including concomitant medications and concurrent ocular procedures), and assessment of safety. BCVA was determined as the logarithm of the minimum angle of resolution (LogMAR).

Exclusion criteria were a history of glaucoma, uveitis, retinal diseases other than CRVO, diabetes mellitus, rubeosis iridis, ocular infections, laser photocoagulation, and intraocular surgery (including cataract surgery).

### Changes of clinical parameters

To assess improvement of vision, the change of BCVA was calculated by subtracting the value after IRI from the value before IRI. To assess improvement of macular edema, the percent change of CMT (%ΔME) was calculated as follows: %ΔME = (1-ME_post_/ME_pre_) × 100 where ME_pre_ and ME_post_ were the levels of macular edema (ME) corresponding to the CMT before IRI and 1 month after IRI, respectively.

### Statistical analysis

Data are presented as the mean with ±standard deviation, as the median with interquartile range, or as frequencies. The paired *t*-test was employed to compare continuous variables between baseline and 1 month or 6 months after IRI, as well as between before and after the 1st recurrence. To examine relationships among the variables, Pearson’s correlation coefficients were calculated. Statistical significance was considered at *P* < 0.05.

## Results

The CRVO patients included 13 men and 7 women aged 67.4 ± 13.1 years (mean ± SD). The mean duration of CRVO was 47.9 ± 62.2 days (range: 9–270 days). Fifteen patients (75%) had hypertension and 7 patients (35%) had hyperlipidemia. Mean baseline BCVA was logMAR 0.53 ± 0.49 and BCVA improved significantly to logMAR 0.25 ± 0.41 at 6 months after IRI. Mean baseline CMT was 689 ± 235 μm and it showed a significant decrease to 414 ± 187 μm after 6 months. At 6 months after IRI, the mean number of recurrent episodes of macular edema was 1.6 ± 1.1 (no recurrence in 4 patients, 1 in 5 patients, 2 in 6 patients, and 3 in 5 patients).

**Fig. 1:**
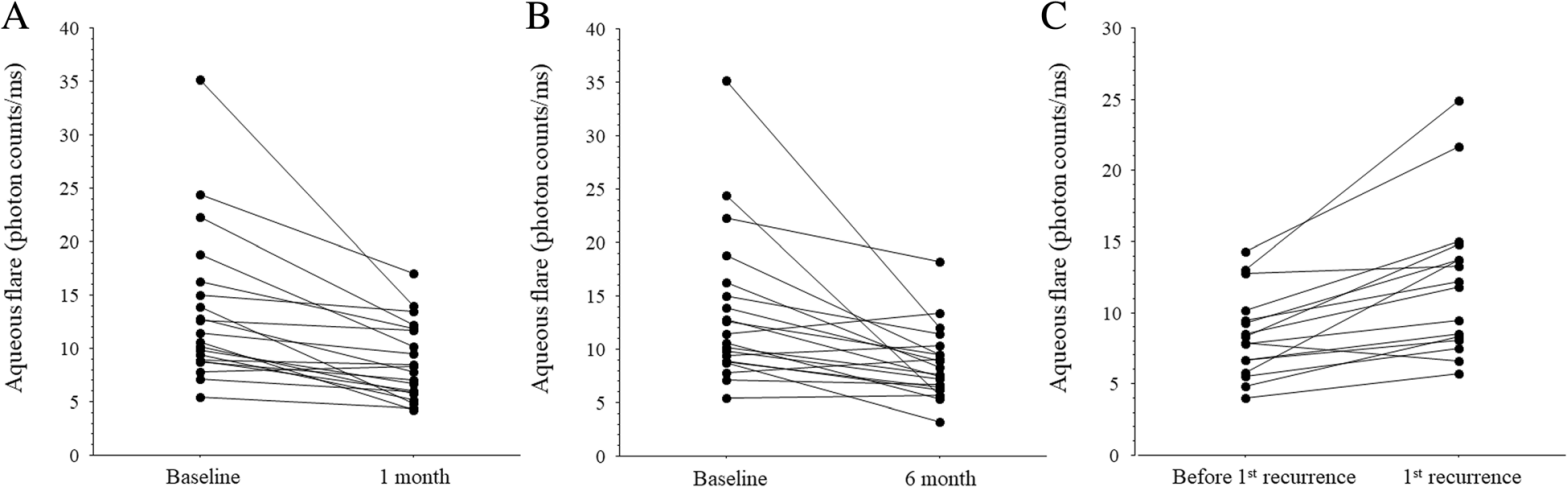
Aqueous flare value at 1 and 6 months after intravitreal ranibizumab injection (IRI) and at the 1st recurrence of macular edema. The aqueous flare value was significantly lower than the baseline value (13.5 ± 7.1photon counts/ms) at (**a**) 1 month after IRI (8.7 ± 3.6 photon counts/ms) and at (**b**) 6 months after IRI (8.6 ± 3.4 photon counts/ms) (*P* < 0.001 and *P* = 0.003, respectively). **c** In the 16 patients with recurrence, the aqueous flare value was significantly higher at the 1st recurrence (12.2 ± 5.3 photon counts/ms) compared with before recurrence (8.4 ± 3.0 photon counts/ms, *P* < 0.001)

The baseline aqueous flare value was 13.5 ± 7.1 photon counts/ms, and it showed a significant decrease at both 1 month (8.7 ± 3.6 photon counts/ms) and 6 months (8.6 ± 3.4 photon counts/ms) after IRI therapy (*P* < 0.001 and *P* = 0.003, respectively) (Figs. 1a and b). In the 16 patients with recurrence, the aqueous flare value was significantly higher at the time of the 1st recurrence (12.2 ± 5.3 photon counts/ms) than before the 1st recurrence (8.4 ± 3.0 photon counts/ms, *P* < 0.001) (Fig. 1c).

Table 1 shows the aqueous humor levels of growth factors/receptors and inflammatory factors/cytokines (sVEGFR-1, sVEGFR-2, VEGF, PlGF, PDGF-AA, sICAM-1, MCP-1, IL-6, IL-8, IL-12 (p70), and IL-13) at baseline and at the 1st recurrence. The median baseline aqueous humor level of sVEGFR-1, sVEGFR-2, VEGF, PlGF, PDGF-AA, sICAM-1, MCP-1, IL-6, IL-8, IL-12(p70), and IL-13 was 1050 pg/ml, 512 pg/ml, 56.7 pg/ml, 4.63 pg/ml, 20.0 pg/ml, 8.45 ng/ml, 2191 pg/ml, 12.2 pg/ml, 30.6 pg/ml, 1.42 pg/ml, and 0.49 pg/ml, respectively. The median aqueous humor level of sVEGFR-1, sVEGFR-2, VEGF, PlGF, PDGF-AA, sICAM-1, MCP-1, IL-6, IL-8, IL-12(p70), and IL-13 at the 1st recurrence was 758 pg/ml, 554 pg/ml, 49.9 pg/ml, 4.89 pg/ml, 20.4 pg/ml, 12.3 ng/ml, 1454 pg/ml, 8.51 pg/ml, 14.8 pg/ml, 2.19 pg/ml, and 0.64 pg/ml, respectively.

**Table 1:** Aqueous humor levels of factors/cytokines at baseline and at the first recurrence

Aqueous factors/Cytokines	sVEGFR-1 (pg/ml)	sVEGFR-2 (pg/ml)	VEGF (pg/ml)	PlGF (pg/ml)	PDGF-AA (pg/ml)	sICAM-1 (ng/ml)	MCP-1 (pg/ml)	IL-6 (pg/ml)	IL-8 (pg/ml)	IL-12(p70) (pg/ml)	IL-13 (pg/ml)
Baseline (median [interquartile range])	1050 [535–1613]	512 [394–809]	56.7 [0.64–110]	4.63 [1.33–10.8]	20.0 [13.7–40.2]	8.45 [2.26–28.5]	2191 [943–3091]	12.2 [2.69–21.9]	30.6 [13.6–48.7]	1.42 [0.18–3.21]	0.49 [0.12–3.34]
1st Recurrence (median [interquartile range])	758 [225–1114]	554 [364–759]	49.9 [0.64–72.6]	4.89 [2.10–8.55]	20.4 [16.0–27.2]	12.3 [1.81–18.9]	1454 [1127–1918]	8.51 [0.64–17.2]	14.8 [7.66–24.8]	2.19 [0.24–2.93]	0.64 [0.16–2.09]

*sVEGFR* soluble vascular endothelial growth factor receptor, *VEGF* vascular endothelial growth factor, *PlGF* placental growth factor, *PDGF* platelet-derived growth factor, *sICAM* soluble intercellular adhesion molecule, *MCP* monocyte chemotactic protein, *IL* interleukin

At baseline, significant correlations were noted between the aqueous flare value and the aqueous humor levels of sVEGFR-1, VEGF, PlGF, PDGF-AA, sICAM-1, MCP-1, IL-6, and IL-8 (Table 2). In contrast, there were no significant correlations between the aqueous flare value and aqueous humor levels of sVEGFR-2, IL-12, or IL-13 (Table 2). In the 16 patients with recurrence, there were also significant correlations between the aqueous flare value and aqueous humor levels of sVEGFR-1, VEGF, PlGF, IL-6, and IL-8 at the 1st recurrence (Table 2), while there were no significant correlations between the aqueous flare value and aqueous humor levels of sVEGFR-2, PDGF-AA, sICAM-1, MCP-1, IL-12, or IL-13 (Table 2).

**Table 2:** Correlations between aqueous humor factors/cytokines and aqueous flare values at baseline and at the first recurrence

Aqueous factors/Cytokines	sVEGFR-1	sVEGFR-2	VEGF	PlGF	PDGF-AA	sICAM-1	MCP-1	IL-6	IL-8	IL-12(p70)	IL-13
Variable	*r* *P* value	*r* *P* value	*r* *P* value	*r* *P* value	*r* *P* value	*r* *P* value	*r* *P* value	*r* *P* value	*r* *P* value	*r* *P* value	*r* *P* value
Aqueous flare at Baseline	0.71 < 0.001	0.33 0.151	0.70 < 0.001	0.45 0.043	0.49 0.033	0.50 0.031	0.58 0.011	0.47 0.035	0.67 0.003	0.11 0.627	0.10 0.659
Aqueous flare at 1st Recurrence	0.51 0.047	0.42 0.097	0.67 0.005	0.51 0.041	0.14 0.592	−0.05 0.849	0.36 0.166	0.59 0.022	0.71 0.002	0.32 0.203	0.36 0.170

*sVEGFR* soluble vascular endothelial growth factor receptor, *VEGF* vascular endothelial growth factor, *PlGF* placental growth factor, *PDGF* platelet-derived growth factor, *sICAM* soluble intercellular adhesion molecule, *MCP* monocyte chemotactic protein, *IL* interleukin, *r* = correlation coefficient. Spearman’s rank-order correlation coefficients were calculated

**Fig. 2:**
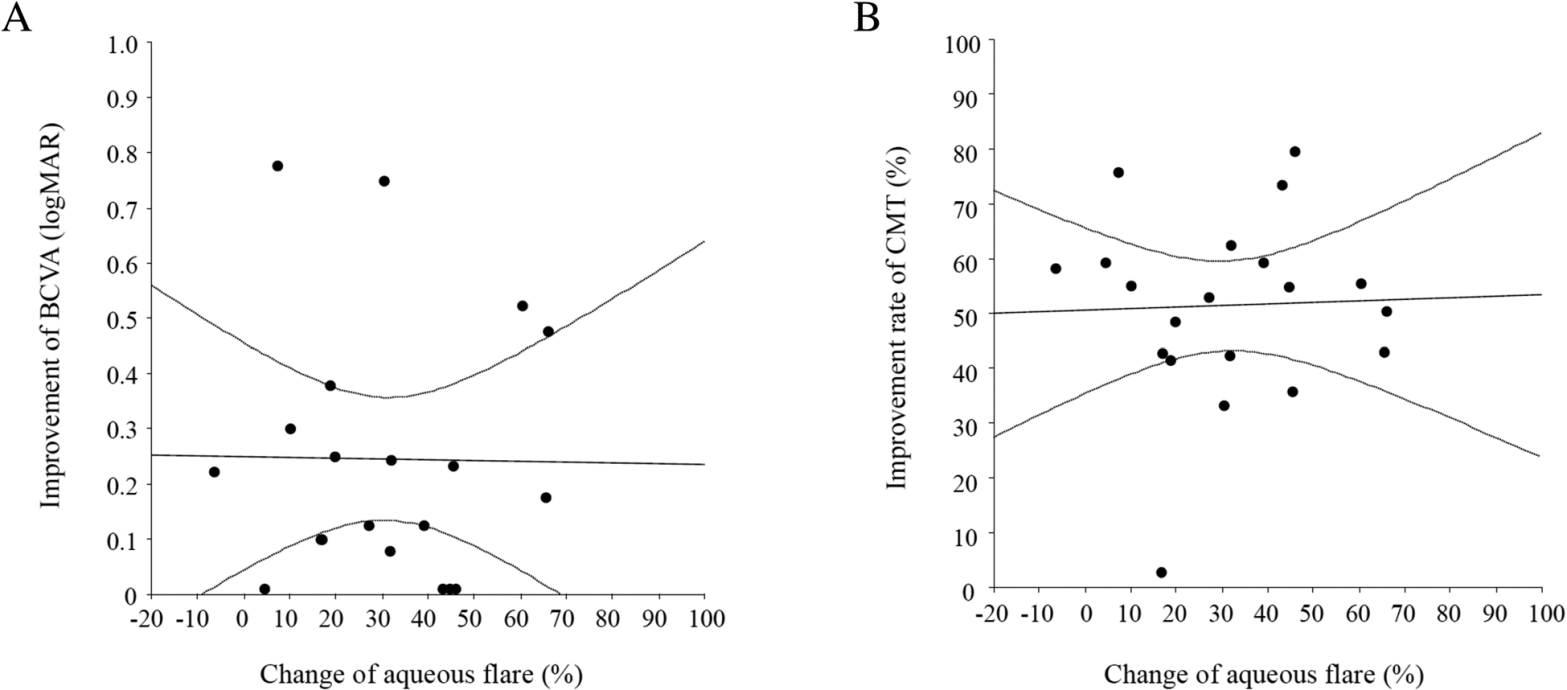
Correlation between the change of the aqueous flare value and improvement of BCVA or CMT at 1 month after intravitreal ranibizumab injection (IRI). **a** There was no significant correlation between the change of the aqueous flare value and improvement of BCVA at 1 month after IRI (ρ = − 0.01, *P* = 0.952). **b** There was also no significant correlation between the change of the aqueous flare value and improvement of CMT at 1 month after IRI (ρ = 0.03, *P* = 0.887)

**Fig. 3:**
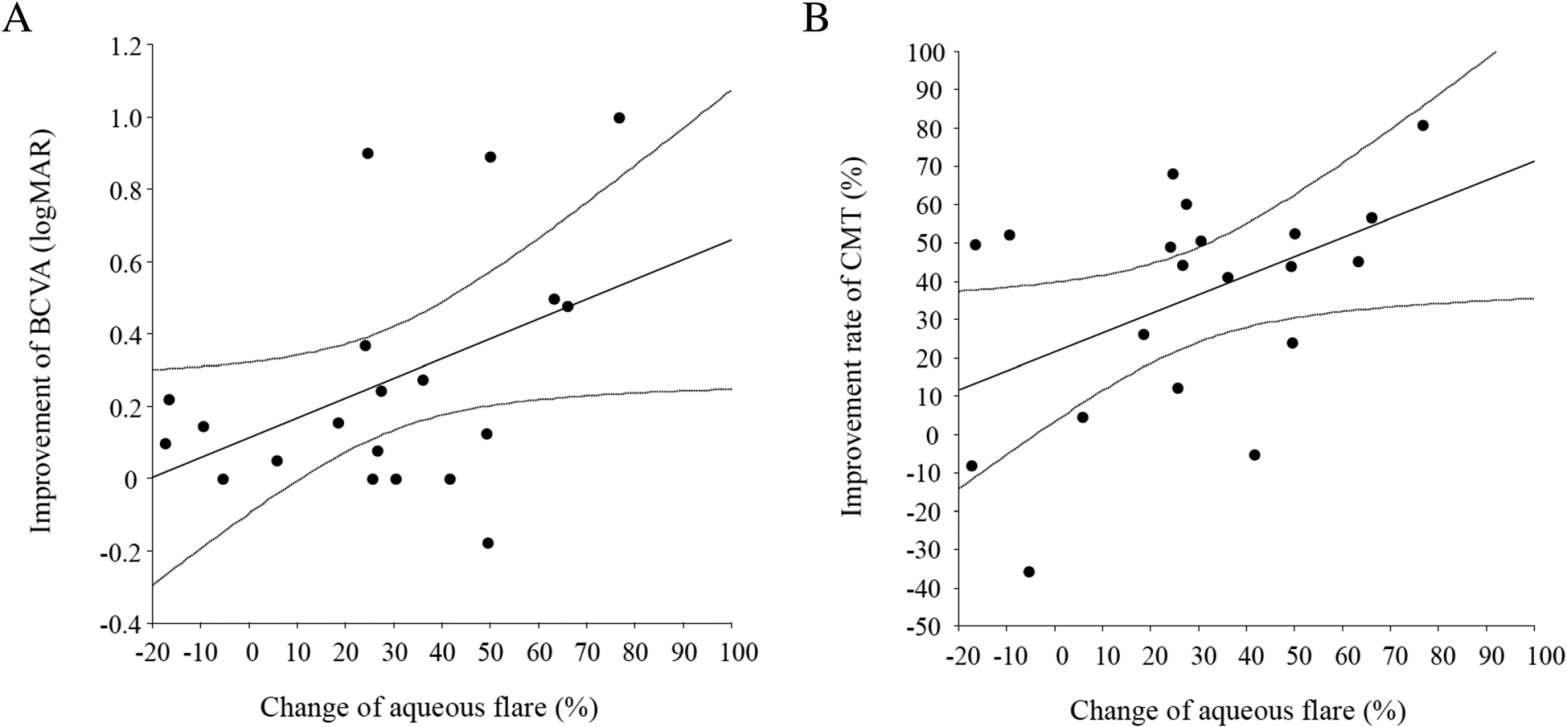
Correlation between the change of the aqueous flare value and improvement of BCVA or CMT at 6 months after intravitreal ranibizumab injection (IRI). **a** There was a significant positive correlation between the change of the aqueous flare value and improvement of BCVA at 6 months after IRI (ρ = 0.45, *P* = 0.047). **b** There was also a significant positive correlation between the change of the aqueous flare value and improvement of CMT at 6 months after IRI (ρ = 0.47, *P* = 0.038)

**Fig. 4:**
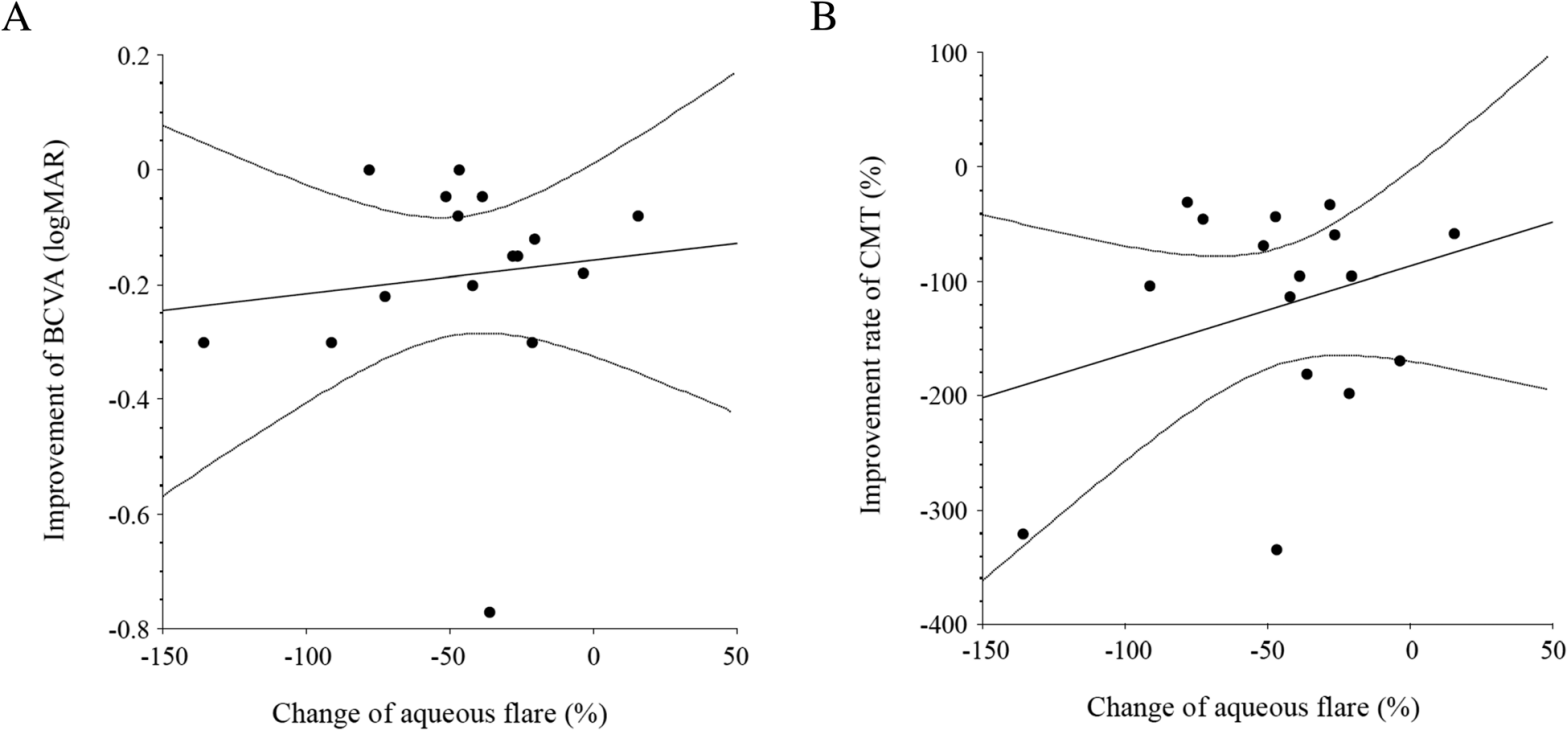
Correlation between the change of the aqueous flare value and improvement of BCVA or CMT at the 1st recurrence after intravitreal ranibizumab injection (IRI). **a** There was no significant correlation between the change of the aqueous flare value and improvement of BCVA at the 1st recurrence after IRI (ρ = 0.11, *P* = 0.673). **b** There was also no significant correlation between the change of the aqueous flare value and improvement of CMT at the 1st recurrence after IRI (ρ = 0.29, *P* = 0.276)

There was no significant correlation between changes of the aqueous flare value and improvement of BCVA or CMT at 1 month after IRI therapy (ρ = − 0.01, *P* = 0.952; ρ = 0.03, *P* = 0.887) (Fig. 2a and b). On the other hand, a significant correlation was identified between the change of the aqueous flare value and improvement of BCVA at 6 months (ρ = 0.45, *P* = 0.047) (Fig. 3a), as well as between the change of the aqueous flare value and the decrease of CMT at 6 months (ρ = 0.47, *P* = 0.038) (Fig. 3b). However, the change of the aqueous flare value was not significantly correlated with improvement of BCVA or CMT at the 1st recurrence (ρ = 0.11, *P* = 0.673; ρ = 0.29, *P* = 0.276) (Fig. 4a and b).

## Discussion

In the present study, there was a significant decrease of the aqueous flare value at both 1 month and 6 months after initiation of IRI therapy for macular edema in patients with CRVO. This suggests that IRI reduces inflammation associated with CRVO. Campochiaro et al. recently proposed a positive feedback loop for VEGF [7], in which vascular occlusion causes retinal ischemia and ischemic retinal cells release VEGF, leading to exacerbation of retinal nonperfusion by the promotion of leukostasis, a change that is associated with inflammation. IRI interrupts this positive feedback loop because it neutralizes VEGF, so the aqueous flare value may have decreased in our CRVO patients following suppression of inflammation. In this study, we also demonstrated a significant positive correlation between the aqueous flare value and the aqueous humor levels of sVEGFR-1, growth factors (VEGF, PlGF, and PDGF-AA), and inflammatory factors/cytokines (sICAM-1, MCP-1, IL-6, and IL-8), indicating that ocular levels of these factors/cytokines are elevated in patients with higher flare values. Binding of VEGF and PlGF to VEGFR-1 induces chemotaxis of leukocytes and promotes inflammation [8–10], while binding of VEGF to VEGFR-2 upregulates the expression of inflammatory factors such as MCP-1 and ICAM-1 via nuclear factor-kappa B [11–13]. Thus, chemotaxis and leukocyte adhesion are promoted by VEGF and PlGF, leading to the onset of inflammation, suggesting that the aqueous flare value may be increased by protein leakage from iridal vessels after disruption of the blood-aqueous barrier by these factors. IRI therapy may inhibit various inflammatory factors by suppression of signaling through VEGFR-1 and -2, resulting in a decrease of the aqueous flare value.

In this study, a significant correlation was identified between the change of the aqueous flare value and improvement of CMT at 6 months after IRI therapy. As mentioned above, IRI decreases the aqueous flare and it also reduces vascular permeability due to suppression of VEGF [7], explaining why the change of the aqueous flare value was significantly correlated with the improvement of CMT at 6 months. However, there was no significant correlation between the change of the aqueous flare value and improvement of CMT at 1 month after IRI therapy, suggesting that the pathological mechanism may not have been suppressed in the early period after administration. Based on this result, it may be important to intensify early treatment for macular edema in CRVO patients. For example, administration of multiple doses may be preferable during the induction phase of therapy or the dosage of the anti-VEGF agent could be increased.

Interestingly, we found a significant positive correlation between the change of the aqueous flare value and improvement of BCVA at 6 months after IRI, although there was no such correlation at 1 month. Therefore, it seems that visual acuity gradually improved at 6 months after IRI therapy along with reduction of CMT, indicating that recovery of visual acuity is a slow process. In addition, the change of the aqueous flare value could be an index of the long-term prognosis in CRVO patients receiving IRI therapy for macular edema.

In this study, 16/20 patients showed recurrence by 6 months after initiation of IRI therapy. In these 16 patients, the aqueous flare value was significantly higher at the 1st recurrence than before recurrence, suggesting that active inflammation can recur in CRVO patients with macular edema after being suppressed by IRI. This is probably because the effect of IRI decreases over time and allows inflammation to develop again via reactivation of the positive feedback loop for VEGF [7], resulting in elevation of the aqueous flare value due to increased production of various inflammatory factors and cytokines. Support for this mechanism was provided by the significant correlations between the aqueous flare value and aqueous humor levels of various inflammatory factors at the 1st recurrence after IRI. Accordingly, an increase of the aqueous flare value may suggest a higher possibility of recurrence after IRI in CRVO patients with macular edema.

## Conclusions

In conclusion, aqueous humor levels of inflammatory factors/cytokines were significantly correlated with the aqueous flare value at baseline and at the 1st recurrence after IRI therapy for macular edema. In addition, the aqueous flare value was significantly lower than the baseline value at both 1 and 6 months after IRI, with a significant correlation being identified between the change of the aqueous flare value and improvement of CMT or BCVA at 6 months. These findings suggest that the aqueous flare value is associated with the ocular levels of inflammatory factors/cytokines, and that the change of this value may be an indicator of the long-term prognosis in CRVO patients receiving IRI therapy for macular edema.

## Abbreviations

BCVA: Best-corrected visual acuity
CMT: Central macular thickness
CRVO: Central retinal vein occlusion
IL: Interleukin
IRI: Intravitreal ranibizumab injection
LogMAR: Logarithm of minimal angle of resolution
MCP-1: Monocyte chemoattractant protein 1
PDGF: Platelet-derived growth factor
PlGF: Placental growth factor
sICAM-1: Soluble intercellular adhesion molecule-1
VEGF: Vascular endothelial growth factor
VEGFR: VEGF receptor
